# Association of Family-Centered Care With Psychological Distress Among Caregivers of Children With Cancer at a Tertiary-Level Hospital in Ethiopia: Cross-Sectional Study

**DOI:** 10.2196/54715

**Published:** 2024-10-10

**Authors:** Leul Deribe, Eshetu Girma, Nataliya Lindström, Abdulkadir Gidey, Solomon Teferra, Adamu Addissie

**Affiliations:** 1 School of Nursing and Midwifery College of Health Sciences Addis Ababa University Addis Ababa Ethiopia; 2 School of Public Health College of Health Sciences Addis Ababa University Addis Ababa Ethiopia; 3 Department of Applied Information Technology University of Gothenburg Goteborg Sweden; 4 School of Medicine College of Health Sciences Addis Ababa University Addis Ababa Ethiopia

**Keywords:** child cancer, psychological distress, Ethiopia, parent, caregivers, family

## Abstract

**Background:**

Psychological distress (PD) is a common mental health problem faced by caregivers of children with cancer. The involvement of families in childcare was found to be associated with lower levels of distress.

**Objective:**

The study aims to determine the associations between family-centered care (FCC) and PD among caregivers of children with cancer receiving treatment at Tikur Anbessa Specialized Hospital (TASH), Ethiopia.

**Methods:**

An institution-based, cross-sectional study was conducted from June to December 2022. Caregivers of children with cancer aged 0-14 years receiving cancer treatment at the pediatric oncology unit completed a face-to-face, interviewer-administered, structured questionnaire during a routine inpatient or outpatient visit. The questionnaire included questions on the characteristics of the child and caregiver, PD (measured by the Kessler Psychological Distress Scale [K10]), FCC (measured by the Measure of Processes of Care [MPOC-20]), and social support (measured by the Oslo-3 Social Support Scale [OSS-3]). Data were collected using the Kobo toolbox and exported to SPSS (version 26; IBM Corp) for cleaning and analysis. A multivariable logistic regression model was used. An odds ratio with a 95% CI was calculated, and a *P* value less than .05 was considered statistically significant.

**Results:**

A total of 384 caregivers of children with cancer participated in the study. The total PD score ranged from 10 to 50, with a mean score of 17.30 (SD 8.96; 95% CI 16.84-18.60). The proportion of caregivers found to have mild, moderate, and severe levels of PD was 43 (11.2%), 35 (9.1%), and 51 (13.3%), respectively. The overall prevalence of mild to severe PD symptoms was 33.6% (95% CI 28.9%-38.3%). A statistically significant negative association was found between FCC and PD (adjusted odds ratio [AOR] 0.68, 95% CI 0.53-0.86). In addition, having no formal education (AOR 2.87, 95% CI 1.28-6.45), having a history of relapse (AOR 3.24, 95% CI 1.17-9.02), beginning cancer treatment at TASH (AOR 2.82, 95% CI 1.4-4.85), beginning treatment within the last 3 months (AOR 3.99, 95% CI 1.73-9.23), and beginning treatment within the last 4 to 18 months (AOR 2.68, 95% CI 1.25-5.76) were significantly associated with higher level of PD.

**Conclusions:**

A total of 1 in 3 caregivers have reported PD. FCC was found to be protective of PD. The finding of this study suggests the need for FCC intervention to improve the mental health condition of caregivers. In addition, the intervention needs to consider the educational status of the caregivers, the time since the cancer diagnosis, and the history of relapse.

## Introduction

A diagnosis of childhood cancer has been described as a life-changing experience for caregivers that causes significant disruptions in a child’s and family’s life. Caregivers face multiple and inescapable stressors associated with their child’s illness, diagnostic procedures, and cancer treatment [1]. Childhood cancer increases the risk of long-term emotional strain [2] and the risk of developing psychosocial distress [3]. Psychological distress (PD) often refers to an undifferentiated combination of symptoms, which is a state of emotional distress characterized by symptoms of depression and anxiety [4]. Distress is defined by the National Comprehensive Cancer Network (NCCN) [5] as follows:

an unpleasant experience of a mental, physical, social, or spiritual nature. This affects the way people think, feel, or act and interferes with the ability to cope with cancer, its physical symptoms, and its treatment.

Caregivers of children with cancer have a higher risk of developing negative emotional states and poor health behaviors [6]. Thus, the prevalence of PD was higher at the time of diagnosis and remained significant during and even after the end of treatment [3,7,8]. It was also found that PD universally affects caregivers of children with cancer. In the United States, 50% of pediatric cancer caregivers are highly distressed and 16% meet the criteria for serious PD [8]. In the United Kingdom, 66% of caregivers of children with cancer have PD [9]. Similarly, 56.0% and 70.5% of caregivers in Lebanon [10] and in Iraq [11] were depressed, respectively. In African studies, PD was reported by 45% and 66.7% of caregivers of children with cancer in Uganda [12] and Tanzania [13], respectively. According to a study conducted in Ethiopia, 72.4% of caregivers of children with cancer had depression [14].

It has also been indicated that parental PD can be affected by various factors. These factors include prognosis and stage of child cancer, child symptom level, treatment status, side effects of chemotherapy, knowledge about treatment modality, treatment cost, employment status, sex of the parent, number of hospital admissions, and family support [8,15-19]. Studies have reported that family-centered care (FCC), which provides holistic care and is planned around the family, is associated with lower PD [20]. Caregivers receiving FCC have been found to have better parental psychosocial health scores, decreased psychological stress, and higher levels of psychological well-being [21-23]. Improved communication and receiving enough information, which are the main components of FCC, are also indicated to have an association with enhanced levels of psychological health in families of children with cancer [24,25].

Although childhood cancer is becoming a significant health burden in Africa [26] and Ethiopia in particular [26], few studies have explicitly examined the level of PD and underlying factors among caregivers of children with cancer. Research on FCC primarily focuses on defining and surveying families and health care providers, with limited exploration of its relationship with family outcomes [20]. To this end, understanding the degree of association between PD and the level of perceived FCC will help to improve the care provided for caregivers of children with cancer. In addition, better insight into factors associated with PD may help to design and implement supportive interventions for caregivers, which reflect their needs in low-income countries. Accordingly, the objective of this study was to describe PD and examine the association between PD and FCC among caregivers of children with cancer receiving treatment at Tikur Anbessa Specialized Hospital (TASH), Ethiopia.

## Methods

### Study Area and Study Design

This study used a cross-sectional design from June to December 2022. The hospital is situated in Addis Ababa, the capital city of Ethiopia. With more than 800 beds, TASH is the largest tertiary university teaching hospital. The hospital offers the most thorough cancer care with over 120 million people nationwide. Patients with cancer from every region were referred to this hospital. The pediatric oncology unit has 26 beds, along with nurses, residents, hemato-oncologists, and hematopathologists. According to pediatric oncology unit registration, between 500 and 600 new children with cancer get both inpatient and outpatient care each year [27].

### Study Participants

Caregivers of children with cancer aged 0-14 years receiving cancer treatment at the TASH pediatric oncology unit were recruited for the study. Due to the restricted number of patients seen during the study period, we did not precalculate the sample size. Instead, we consecutively invited all caregivers who visited the unit during the study period to participate in the study. Accordingly, 393 caregivers of children with cancer were interviewed in this study. A mother or father was selected if they were available with other family members during data collection. If both mothers and fathers were available, the parent who reported spending more time with the child and more frequently visited the health facility was selected.

The inclusion criteria for this study were caregivers or guardians of children with all types of cancer visiting the TASH pediatric oncology unit, having at least 1 previous visit, attending either an inpatient or outpatient department, and at least 1 month had passed since the child’s diagnosis. The term “caregiver” or “guardian” refers to the person who decides on most things for the child daily, both inside and outside the medical environment. The study excluded caregivers or guardians of children with known mental health problems before their child’s cancer diagnosis. A total of 3 caregivers were excluded because they identified themselves as being in treatment for mental health conditions before knowing their child’s cancer diagnosis and 4 caregivers did not provide complete data for the dependent variable.

### Data Collection

The study used a face-to-face, interviewer-administered, structured questionnaire (see Multimedia Appendix 1). The questionnaire was prepared in English and independently translated into Amharic by 2 bilingual translators. A third reviewer, who provided feedback at a reconciliation meeting, checked the translated version. After obtaining the agreement, another language checker translated the Amharic version into English. Any inconsistencies were corrected, and a final Amharic translation was used to collect the data. The caregivers were recruited to the interview consecutively as they come to the pediatric oncology unit for regular visits. The participants’ responses were instantly entered into a Kobo toolbox data collection tool by the data collector at the time of the interview. The face-to-face interviews were approximately 20 minutes.

### Measurements

#### Overview

The survey included questions regarding caregivers’ sociodemographic characteristics, children’s health status, parental distress, and caregivers’ satisfaction with the treatment received. Information on sociodemographic characteristics was obtained by interviewing caregivers using a standard questionnaire. Data regarding the children’s clinical characteristics were obtained from their medical records using a structured data-retrieving checklist with the assistance of an MSc oncology nurse in the pediatric oncology unit.

#### Kessler Psychological Distress Scale

The Kessler Psychological Distress Scale (K10) is a tool consisting of 10 items originally developed to measure PD [28]. It is made of a 5-point Likert-type scale ranging from 1=none of the time to 5=all of the time. The tool assesses the experience of symptoms of mental distress over the past 30 days. The total score of the tool ranges from 10 to 50. Owing to the lack of a universally accepted method for categorizing K10 scores, various methods were used based on the purpose and context of the study. Considering the absence of a specific threshold in Ethiopia’s setting and our aim to screen caregivers of children with cancer for the likelihood of having a mental disorder, we chose the most commonly used grouping for K10 scores to assess PD in primary health care settings. Therefore, the total score was categorized as 10-19, indicating “likely to be well”; 20-24, “likely to have a mild mental disorder”; 25-29, “likely to have a moderate mental disorder”; and above 30, “likely to have a severe mental disorder” [29-32]. Finally, a score less than or equal to 19 was coded as 0 for no mental disorder, and the presence of likelihood of mental disorder for a score greater than or equal to 20 was coded as 1. The K10 scale has been validated and confirmed to effectively assess PD in Ethiopian settings, with Cronbach α of 0.86 and 0.83 reliability [33,34].

#### Measure of Processes of Care

To evaluate the family-centeredness of care, the Measure of Processes of Care (MPOC-20) was used [35]. MPOC-20 can assess both families’ experiences and perceptions of the family-centeredness of services received. The tool is translated and validated in about 14 languages [36]. The MPOC-20 contains 20 items and five scales and they are (1) enabling and partnership (3 items), (2) providing general information (3 items), (3) providing specific information about the child (5 items), (4) coordinated and comprehensive care for the family and child (4 items), and (5) respectful and supportive care (5 items). Patients respond using a 7-point scale that ranges from “not at all” (score=1) to “to a very great extent” (score=7) [35]. MPOC-20 is a widely used tool in different setups to evaluate the level of FCC. For this study, the Cronbach α was found to be 0.92.

#### Oslo-3 Social Support Scale

The 3 questions of the Oslo-3 Social Support Scale (OSS-3) cover the reported number of close friends, perceived concern, and practical help from others, and the sum score ranges from 3 to 12. Scores 3-7 are considered poor, scores 8-10 as moderate, and scores 11-12 as strong social support [37].

### Data Quality Control

The principal investigator guided the data collection process. A total of 2 nurses with MSc in nursing were recruited as supervisors. Six MPH or MSc holders were used as the data collectors. Data collectors and supervisors were not a part of the childcare team. Both supervisors and data collectors received training before data collection. During the training, the need for confidentiality and privacy was emphasized. Different pediatric oncology units at St. Paul Millennium Medical College were used as pretest sites.

### Data Processing and Analysis

The data were collected using the Kobo toolbox data collection tool and exported to SPSS (version 26). Missing values and outliers were checked and corrected. Descriptive statistics were calculated, and the data were presented as tables, graphs, and frequencies. Variables such as family size, number of children in the household, child’s age, duration in months since the child fell ill, and duration in months since the child was diagnosed with cancer were categorized using the first and third quartiles. The strength of the association between the independent and dependent variables was described using the odds ratio with 95% CI. All variables with *P* values less than .20 during binary logistic regression were considered candidates for the final model. In addition, we considered the clinical relevance of the variables in the multivariate model. The final fitted multivariable logistic regression model was constructed using the enter method. Variables with *P* values less than .05 were regarded as having a statistically significant association.

### Ethical Considerations

Ethical clearance was obtained from the Addis Ababa University College of Health Science institutional review board (protocol 022/22/SPH). Permission was also obtained from the TASH pediatric oncology unit. Written informed consent for the interviewee was obtained from each study participant. A private place was used for the interview session to keep the respondents’ privacy. Participants were assured of their right to withdraw from the interview at any time, and participation in this study or refusal to participate would not affect their ability to access health services or any other services. Names and other personal information, which can violate the confidentiality of the study participants, were not recorded. In addition, no compensation was paid to the interviewed participants.

## Results

### Sociodemographic Characteristics of Caregivers

A total of 393 caregivers of children with cancer were approached and 384 caregivers provided complete responses (response rate of 97.7%). The caregivers’ ages ranged from 18 to 75 years, with a median of 35.17 (IQR 28-40) years; 176 (45.8%) were fathers. From 384 caregivers, the majority of the participants were from urban areas (n=233, 60.7%), attended secondary school (n=135, 35.2%), and were employed (n=232, 60.4%). Most of the families (n=350, 91.1%), were married or living together and 267 (30.5%) had a family size of 5 people or more. A total of 64% (248) of the families had 2 to 4 children. The number of caregivers who stated they could save money was 53 (13.8%). Table 1 provides a detailed overview of the participants.

**Table 1:** Sociodemographic characteristics of caregivers of children with cancer at the Tikur Anbessa Specialized Hospital pediatric oncology unit from June to December 2022 (n=384).

Variables and category			Values, n (%)
**Caregivers’ gender**			
	Mother	158 (41.1)	
	Father	176 (45.8)	
	Others^a^	50 (13)	
**Sex**			
	Male	205 (53.4)	
	Female	179 (46.6)	
**Age of the caregiver or guardian (years)**			
	≤29	106 (27.6)	
	30-39	161 (41.9)	
	≥40	117 (30.5)	
**Place of residence**			
	Urban	233 (60.7)	
	Rural	151 (39.3)	
**Employment status**			
	Housewife	90 (23.4)	
	Currently not employed	62 (16.1)	
	Currently employed	232 (60.4)	
**Caregiver or guardian’s educational level**			
	No formal education	62 (16.1)	
	Primary education (grades 1 to 8)	88 (22.9)	
	Secondary education (grades 9 to 12)	135 (35.2)	
	College and above	99 (25.8)	
**Marital status**			
	Currently married or cohabiting	350 (91.1)	
	Not married or not cohabiting	34 (8.9)	
**Family size**			
	≤4 family size	117 (30.5)	
	≥5 family size	267 (69.5)	
**Number of children**			
	Single child	56 (14.6)	
	2-4 children	248 (64.6)	
	≥5 children	80 (20.8)	
**Looking after siblings at home**			
	Yes	288 (75)	
	No	19 (4.9)	
	No one is left at home	77 (20.1)	
**Income and household expenses^b^**			
	Your household can save money	53 (13.8)	
	Your household spends what it earns	215 (56)	
	Your household eats into its assets and savings	68 (17.7)	
	Your household gets into debt	48 (12.5)	

^a^Grandparent, brother, sister, uncle, and aunt.

^b^The variable was intended to assess the income and expenses of a household, and it is adapted from the Swiss Household Panel survey questionnaire [38].

### Social Support

The OSS-3 was used to evaluate the extent of social support among caregivers of children with cancer, with 381 participants offering comprehensive responses. Among them, 282 (72.5%) indicated poor social support, 98 (25.2%) reported moderate support, and 1 caregiver reported high social support.

### Characteristics of Children

Regarding characteristics of children, from the 384 children receiving treatment, the majority (n=227, 59.1%) were male and 78 (20.3%) were older than 10 years. The majority (n=251, 65.4%) of the children were referred to TASH, 357 (93.0%) had no history of relapse, and 325 (84.7%) were receiving treatment. Of 325 children receiving treatment, 247 (69.6%) were getting chemotherapy. Leukemia was the most common cancer type (n=115, 29.6%). From 211 staged cancer cases, 62 (16.1%) were stage IV and 28 (7.3%) were stage III. The median time since the child was ill and diagnosed with cancer was 12 and 7 months, respectively (Table 2).

**Table 2:** Characteristics of children receiving cancer treatment at the Tikur Anbessa Specialized Hospital pediatric oncology unit from June to December 2022 (n=384).

Variables and category			Values, n (%)
**Child’s sex**			
	Male	227 (59.1)	
	Female	157 (40.9)	
**Child age (years)**			
	≤4	153 (39.8)	
	5-9	153 (39.8)	
	≥10	78 (20.3)	
**Place child started treatment**			
	In this hospital	133 (34.6)	
	Referred from other hospital	251 (65.4)	
**History of relapse**			
	Yes	27 (7)	
	No	357 (93)	
**Time in months since the child is ill**			
	≤6	103 (26.8)	
	7-25	204 (53.1)	
	≥26	77 (20.1)	
**Time in months since your child is diagnosed with cancer**			
	≤3	104 (27.1)	
	4-18	191 (49.7)	
	≥19	89 (23.2)	
**Child cancer type**			
	Solid cancer	207 (53.9)	
	Hematological	177 (46.1)	
**Type of malignancy**			
	Leukemia	115 (29.9)	
	Wilms’ tumor	64 (16.7)	
	Sarcoma	53 (13.8)	
	Hodgkin’s lymphoma	45 (11.7)	
	Retinoblastoma	32 (8.3)	
	Central nervous system	30 (7.8)	
	Non-Hodgkin lymphoma	9 (2.3)	
	Others	36 (9.4)	
**Cancer stage**			
	Not staged	173 (45.2)	
	Stage I	59 (15.4)	
	Stage II	62 (16.1)	
	Stage III	28 (7.3)	
	Stage IV	62 (16.1)	
**Treatment status**			
	On treatment	325 (84.6)	
	Waiting to start treatment	19 (4.9)	
	Off treatment	40 (10.4)	
**Type of treatment (n=325)**			
	Chemotherapy only	247 (76)	
	Other treatment type	78 (24)	

### Level of Perceived FCC

The detailed results related to caregivers’ perception of the level of FCC were published [38]. Families provided feedback on the MPOC-20, yielding scores ranging from 1.00 to 6.90 and a mean score of 3.71 (SD 1.04). Among the original 5 domains of the MPOC-20, the highest mean score of 4.81 (SD 1.32) was attributed to respectful and supportive care, followed by coordinated and comprehensive care, with a mean score of 4.64 (SD 1.19). The lowest mean scores were recorded for providing specific information (2.33, SD 0.80) and general information (2.70, SD 1.23).

### Caregivers’ PD

The total K10 score reported by caregivers ranged from 10 to 50. It was skewed to the right with a mean of 17.30 (SD 8.96; 95% CI 16.84-18.60) and a median of 14 (IQR 10-23). Of a total of 384 interviewed caregivers, 43 (11.2%) had mild, 35 (9.1%) had moderate, and 51 (13.3%) of the caregivers had severe PD. The overall prevalence of mild to severe PD symptoms was 33.6% (95% CI 28.9-38.3)

### Association of FCC and PD

Logistic regression was used to examine the association of FCC and PD while controlling other variables. During bivariate logistic regression analysis, FCC, place of residence, educational level, level of income and expense, referral from other facilities, history of relapse, time since the child was diagnosed, type of cancer, and treatment status were found to have a significant association with caregivers PD. Variables such as sex of the caregivers, caregiver age, relationship with the child, working status, marital status, child sex, child age, number of children, perceived social support, and family size did not show any association.

The final multiple logistic regression model included 9 variables with *P* values less than .05 from the bivariate logistic regression analysis. The model, as explained between 20.3% (Cox and Snell *R*^2^) and 28.1% (Nagelkerke *R*^2^) of the variance in PD, correctly classified 76.0% of cases. The model fitness test used the Hosmer-Lemeshow statistic, indicating a good model fit (*P*=.46).

After controlling for other variables using multiple logistic regression analysis, FCC was significantly associated with PD. Accordingly, caregivers who reported a unit increase in the level of FCC received were found to reduce PD by 32% (adjusted odds ratio [AOR] 0.68, 95% CI 0.53-0.86). In addition, having no formal education (AOR 2.87, 95% CI 1.28-6.45), having a history of relapse (AOR 3.24, 95% CI 1.17-9.02), starting cancer treatment at TASH (AOR 2.82, 95% CI 1.4-4.85), starting treatment within the last 3 months (AOR 3.99, 95% CI 1.73-9.23), and starting treatment within the last 4 to 18 months (AOR 2.68, 95% CI 1.25-5.76) were significantly associated with higher level of PD (see Table 3).

**Table 3:** Logistic regression analysis for factors affecting the level of psychological distress among caregivers of children with cancer.

Variables or characteristics			Distressed					Crud OR^a^ (95% CI)			Adjusted OR (95% CI)			*P* value
			Yes		No									
Family-centered care (MPOC^b^-20 mean score), mean (SD)			3.45 (1.07)		3.85 (1.01)		0.69 (0.56-0.85)			0.68 (0.53-0.86)			.001	
**Residential place, n (%)**														
	Urban	63 (27)		170 (73)		0.47 (0.31-0.73)			0.73 (0.42-1.25)			.25		
	Rural	66 (43.7)		85 (56.3)		Reference			Reference					
**Educational status, n (%)**														
	No formal education	30 (48.4)		32 (51.6)		3.28 (1.65-6.53)			2.87 (1.28-6.45)			.01		
	Primary education	37 (42.0)		51 (58.0)		2.54 (1.35-4.79)			1.83 (0.87-3.83)			.10		
	Secondary education	40 (29.6)		95 (70.4)		1.47 (0.80-2.69)			1.38 (0.71-2.67)			.35		
	College and above	22 (22.2)		77 (77.8)		Reference			Reference					
**Household income and expenses, n (%)**														
	Household can save money	13 (24.5		40 (75.5)		Reference			Reference					
	Household spends what it earns	69 (32.1)		164 (67.9)		1.45 (0.73-2.89)			1.54 (0.70-3.38)			.29		
	Household eats into its assets and savings	19 (27.9)		49 (72.1)		1.19 (0.53-2.71)			0.70 (0.26-1.87)			.48		
	Household gets into debt	28 (58.3)		20 (41.7)		4.31 (1.84-10.1)			2.35 (0.88-6.29)			.09		
**Child cancer type, n (%)**														
	Solid cancer	81 (39.1)		126 (60.9)		1.73 (1.12-2.67)			1.54 (0.94-2.53)			.09		
	Hematological	48 (27.1		129 (72.9)		Reference			Reference					
**History of relapse, n (%)**														
	Yes	14 (51.9)		13 (51.9)		2.26 (1.03-4.98)			3.24 (1.17-9.02)			.02		
	No	115 (32.2)		242 (32.2)		Reference			Reference					
**Time since diagnosis (months), n (%)**														
	Below 3	47 (45.2)		57 (54.8)		3.49 (1.82-6.72)			3.99 (1.73-9.23)			.001		
	4*-*18	65 (34.0)		126 (66.0)		2.19 (1.19-4.01)			2.68 (1.25-5.76)			.01		
	Above 19	17 (19.1)		72 (80.9)		Reference			Reference					
**Treatment status, n (%)**														
	Waiting or off treatment	11 (18.6)		48 (81.4)		0.40 (0.20-0.80)			0.55 (0.25-1.23)			.15		
	On treatment	118 (36.3)		207 (63.7)		Reference			Reference					
**Place of starting treatment, n (%)**														
	In this hospital	68 (51.1)		65 (48.9)		3.26 (2.09-5.09)			2.82 (1.64-4.85)			<.001		
	Referred from other hospital	61 (24.3)		190 (75.7)		Reference			Reference					

^a^OR: odds ratio.

^b^MPOC: a measure of process of care.

## Discussion

### Principal Findings

In this study, the relationships between the perception of FCC and PD in caregivers of children with cancer visiting inpatient and outpatient pediatric oncology units were examined. It is the first study to investigate the level of PD using a locally validated standard tool and investigate its association with FCC. To our knowledge, no study has focused on PD among caregivers of children with cancer receiving treatment in both inpatient and outpatient settings in Ethiopia. The study also evaluated the effect of factors related to caregivers’ sociodemographic, child health, and health facility–related factors on PD among caregivers of children with cancer. The findings from this study provide information on the burden of PD and identify the factors contributing to PD among caregivers.

Our findings show that 1 in 3 interviewed caregivers reported mild to severe PD, which indicates that parenting a child with cancer can profoundly affect caregivers’ mental health. This finding is higher than the 0.3% mild to severe level of distress reported among the general population scored using the K10 in Ethiopia [33]. This justifies the need to include screening for PD as a critical element of comprehensive psychosocial care for caregivers of children with cancer [39]. This is supported by previous studies that state mental health screening and care for caregivers help to improve child health by facilitating communication among health care providers and caregivers [40].

The level of mild to severe PD in this study was found to be lower than studies conducted in the United States [8], Lebanon [10], Tanzania [13], and Uganda [12]. This difference in the level of PD may be attributed to sociodemographic differences and differences in the measurement tools used. Notably, in this study, unlike in previous studies, most of the participants were fathers. Since the study was conducted in the leading referral hospitals in Ethiopia, the children came from distant parts of the country. Because of this, primarily fathers bring their sick children to hospitals. Multiple studies have reported that fathers have lower levels of parental distress than mothers [41,42]. Another reason might be due to the fact that this study was conducted in a hospital setting where caregivers consider starting treatment as the main achievement in the cancer care process [43]. In Ethiopia, as caregivers have to wait and go through long bureaucracy and referral systems, starting treatment at TASH may have created a sense of satisfaction and increased hope for a cure. This is supported by previous research, which states that beginning treatment is the main reason for lower stress levels [43].

In this study, caregivers’ perceptions of FCC were found to be protective against PD. Our finding is consistent with other studies that reported the association of a higher level of perceived FCC with decreased mental health problems [21,22]. Similarly, previous studies indicated improved psychological health among caregivers who received FCC-based intervention [24,25]. The main components of FCC, such as the provision of specific and general cancer-related information [43,44], enabling caregivers to participate in childcare and decision-making [45,46], having good communication [24,25], and providing coordinated and comprehensive care, were found to decrease caregivers’ PD. FCC also reduced parental distress caused by lack of information [43], underscoring the importance of considering the complex nature of caregivers’ needs and integrating FCC into pediatric oncology care. FCC can be incorporated into child cancer treatment by offering information and education, preparing families before a child’s procedures, enhancing communication between caregivers and the health care team, and providing adequate support and empowerment for caregivers [24,25,49-53].

Our findings show caregivers of children with lower educational levels were more likely to have higher levels of PD than more educated caregivers (college and above), which aligns with previous research [11,16]. Education enables individuals to develop fundamental skills, abilities, and resources for more effective health behaviors, helping them to acquire or create effective means of achieving better health [54,55]. On the contrary, a lower educational level may lead to a higher economic burden, a lower understanding of the information provided to caregivers, and a decreased sense of control over an individual’s surroundings [56,57]. In addition, lower education levels might affect caregivers coping mechanisms; more educated people may be better equipped (have better cognitive skills) to deal with the consequences of childhood cancer [4]. This finding indicates the education level of caregivers needs to be considered when preparing and delivering information and care.

The results of this study also indicated caregivers of children with a history of relapse had higher levels of PD. This is similar to previous studies [58,59]. Caregivers of children with cancer have uncertainties associated with the fear of negative consequences such as relapse or death [60]. The relapse of children with cancer could lead to prolonged uncertainty, negative expectations about disease progression, increased fear of losing their child, and causing poor emotional and mental health [61,62]. Facing the trajectories of childhood cancer for the second time might also be associated with feelings of sadness and frustration, which increases the sense of helplessness, vulnerability, and lack of control over the events [58,62]. All these conditions are associated with adverse mental health conditions.

Furthermore, relapse may be considered a sign of treatment failure, complications, or poor disease progression [63]. This perception may also play a role in higher levels of distress. Consequently, the overwhelming distress of relapse has the potential to compromise families’ information-processing abilities and further increase their level of PD. Despite this, previous studies have reported that caregivers of children with relapse reported lower levels of distress than the onset of child cancer because of caregivers’ ability to learn faster and having prior information about the possibility of relapse [64]. Therefore, establishing a screening mechanism for caregivers of children with relapse, creating good communication, and providing adequate information about the possibility and management of relapse will help to reduce caregivers’ PD.

Another child health–related factor that serves as an independent predictor of PD is the duration since diagnosis. Our study revealed caregivers with a shorter period since diagnosis reported higher levels of PD. Similar findings from previous studies show PD is typically higher during the initial diagnosis and gradually decreases over time [2,3,7,8]. This could be attributed to the highly demanding nature of care provided to children with cancer at the time of diagnosis. Caregivers from low-income countries such as Ethiopia have little or no information about what to do when they first hear their child’s diagnosis. As time passes, caregivers gain more understanding and develop some of the skills required to care for their sick child [43]. Thus, this study indicates the importance of providing well-designed psychosocial support, focusing on the time of diagnosis. Caregivers who started treatment for the first time in their current hospital were also found to have more PD than those referred from other hospitals. According to a qualitative study [43] conducted in a similar setting, hospital-related factors such as long waiting time for both diagnosis and treatment, shortage of chemotherapy drugs, and high patient load might be the reason why patients who started treatment at TASH had higher levels of PD.

### Limitation

This study should be understood in the setting of potential limitations. Because caregivers participated in the study after their child was diagnosed with cancer, the baseline distress level before the diagnosis of their child was not known. To minimize this limitation, caregivers were asked for known mental health conditions and excluded from the study. But still, it was not possible to identify subclinical and undiagnosed cases before a diagnosis of child cancer. Because of the cross-sectional nature of the study design, it is impossible to formulate cause-and-effect relationships among variables. It will be necessary for follow-up studies to include more detailed relationships among variables. Finally, social desirability bias might be introduced since we conducted interview-based surveys.

### Conclusions and Recommendation

In this study, the mean K10 was 17.30 (SD 8.96). A total of 1 in 3 caregivers has reported mild to severe levels of PD. Receiving a higher level of FCC was found to be protective for parental PD. In addition, lower educational status, history of relapse, and shorter time since diagnosis and starting treatment at TASH were associated with higher levels of PD. The results suggest that screening for PD following a child’s diagnosis may help to identify distressed caregivers early and potentially lead to earlier psychosocial intervention. Furthermore, in developing evidence-based interventions for caregivers of ill children, it is important to understand the potential risk factors for increased parental distress. The risk factors associated with PD found in this study suggest that interventions need to address the needs of lower socioeconomic conditions, caregivers who had children with relapse, and during the early stage of child diagnosis. Furthermore, conducting further studies, including siblings, will help to get more detailed insight into the mental health effects of childhood cancer at the family level.

## Appendix

Multimedia Appendix 1 Structured questionniare.

## Abbreviations

AOR: adjusted odds ratio
FCC: family-centered care
K10: Kessler Psychological Distress Scale
MPOC-20: Measure of Processes of Care
NCCN: National Comprehensive Cancer Network
OSS-3: Oslo-3 Social Support Scale
PD: psychological distress
TASH: Tikur Anbessa Specialized Hospital

## Data Availability

All data generated or analyzed during this study are included in this published article.
