# Supplementary material for: Association of Family-Centered Care With Psychological Distress Among Caregivers of Children With Cancer at a Tertiary-Level Hospital in Ethiopia: Cross-Sectional Study
Source: JMIR Cancer. 2024 Oct 10;10:e54715. doi: 10.2196/54715 (PMC11502982; doi:10.2196/54715)
Supplement: Multimedia Appendix 1 [file cancer_v10i1e54715_app1.docx]

Questionnaire prepared for researching on level of parental psychological stress among parents of children with cancer and family centeredness of care at Tikur Anbessa specialized hospital, Ethiopia.

|  | **SECTION 1 Parent Characteristics** | | | | | | | | | | | | |
| --- | --- | --- | --- | --- | --- | --- | --- | --- | --- | --- | --- | --- | --- |
| QUESTIONS & FILTERS | | CODING CATEGORIES | | | | | | | | | | | Skip to |
| If you don’t mind, I would like to start by asking you a little about yourself and your area. | | | | | | | | | | | | |  |
|  | Caregiver’s sex | Female 0  Male 1 | | | | | | | | | | |  |
|  | What is your relationship with the child? | Mother 0  Father 1  Grandmother/father 2  Brother 3  Sister 4  Other (specify) __________________ 5 | | | | | | | | | | |  |
|  | How old were you on your last birthday? | Age (years) ................................ [ ][ ] | | | | | | | | | | |  |
|  | What is your religion? | No religion 0  Orthodox 1  Islam 2  Protestant 3  Catholic 4  Other (specify) _______________________ 5 | | | | | | | | | | |  |
|  | To which ethnic group do you belong? | Oromo 1  Amhara 2  Gurage 3  Tigre 4  Others (specify_________________) 5 | | | | | | | | | | |  |
|  | What is the highest level of education that you achieved? | Illiterate (can’t read and write) 1  Primary (1-6 grade) 2  Primary (7-8 grade) 3  Secondary (9-10 grade) 4  Secondary (11-12 grade) 5  College and above 4 | | | | | | | | | | |  |
|  | Current work condition?  SPECIFY KIND OF WORK | House wife 1  Looking for work/unemployed 2  Student 3  Government employee 4  Private employee 5  Farmer 6  Work at his own firm 7  Merchant 8  *Daily laborer* 9  Retired 10  Other (specify): ________________________ 11 | | | | | | | | | | |  |
|  |  |  |  |  |  |  |  |  |  |  |  |  |  |
|  | Where is your residential place? | Urban 1  Rural 2 | | | | | | | | | | |  |
|  | What is your current marital status? | Not currently married or living with a partner 1  Living with partner, not married 3  Currently having a regular partner, living apart  *4*  Currently married 2  Divorced 5  Separated/broken up 6  Widowed/partner died 7 | | | | | | | | | | |  |
|  | What is your family size? Number of individuals permanently living in your household. (Crowding index) | Family size [ ] [ ] | | | | | | | | | | |  |
|  | Total number of children in the house | Number of children [ ] [ ] | | | | | | | | | | |  |
|  | Is there anyone who is looking after family members left at home | Yes 1  No 2  No one is left at home 3 | | | | | | | | | | | - 116 - 116 |
|  | If yes who is looking for your child at home? | Mother/father 1  Grandmother/father 2  Older sister/brother 3  Housemaid 4  Neighbors 5  Other relatives 6  Other (specify) ­­­­­­­____________________ 7 | | | | | | | | | | |  |
|  | How much do you relay on the relatives caring for your family members at home | Very much 1  A little 2  None 3 | | | | | | | | | | |  |
|  | Do you have frequent contact with religious leaders | Yes 1  No 2 | | | | | | | | | | |  |
|  | Regarding expense of your income and expenses  From provided choices select a statement that best describe your houses use of income | Your household can save money 1  Your household spends what it earns 2  Your household eats into its assets and savings 3  Your household gets into debt 4  No answer / does not know 5 | | | | | | | | | | |  |
|  | Average monthly income | _____________ | | | | | | | | | | |  |
| **LEVEL OF SOCIAL SUPPORT AND RELATIONSHIP (oslo-3 social support scale)** | | | | | | | | | | | | | |
|  | How many people are so close to you that you can count on them if you have any serious personal problems related to your child’s health problem and treatment [choose one option]? | None 1  1 or 2 2  3-5 3  More than 5 4 | | | | | | | | | | |  |
|  | How much concern do people show for what you are doing [choose one option]? | A lot of concern and interest 1  Some concern and interest 2  Uncertain 3  Little concern and interest 4  No concern and interest 5 | | | | | | | | | | |  |
|  | How easy is it to get practical help related to your child’s health problem and treatment from neighbours if you should need it [choose one option]? | Very difficult 1  Difficult 2  Possible 3  Easy 4  Very easy 5 | | | | | | | | | | |  |
| **Child Characteristics** | | | | | | | | | | | | | |
|  | How old is your child’s in his/her last birthday? | AGE (YEARS) ................................ [ ][ ]  Months (if less than one year) [ ][ ] | | | | | | | | | | |  |
|  | Your child’s sex | Female 0  Male 1 | | | | | | | | | | |  |
|  | Cancer type | CNS 1  Leukemia 2  Lymphoma 3  Sarcoma 4  Lung and kidney 5  Other __________________________ 6 | | | | | | | | | | |  |
|  | Cancer stage | Stage I 1  Stage II 2  Stage III 3  Stage IV 4 | | | | | | | | | | |  |
|  | Treatment status | On treatment 1  Waiting to start treatment 2  Off treatment 3 | | | | | | | | | | | - 208 - 208 |
|  | Type of treatment (more than one option is possible) | Chemotherapy 1  Surgery 2  Radiotherapy 3  Others _________________________ 4 | | | | | | | | | | |  |
|  | Time in months since your child is sick with current health problem | Months [ ][ ] | | | | | | | | | | |  |
|  | Time in months since your child is diagnosed with cancer. | Months [ ][ ] | | | | | | | | | | |  |
|  | Time in months since your child started cancer treatment | Months [ ][ ] | | | | | | | | | | |  |
|  | Time since your child is hospitalized | Days [ ][ ] | | | | | | | | | | |  |
|  | Does your child have any history of relapse? | Yes 1  No 2 | | | | | | | | | | |  |
| The Kessler Psychological Distress Scale (K-10)  about how often did you feel… | | | | | | | | | | | | | |
|  | 0 None of the time 1 2 3 4 All of the time | | | | | | | | | | | | |
|  | tired out for no good reason? | | | | 0 | | 1 | | 2 | | 3 | 4 | |
|  | nervous? | | | | 0 | | 1 | | 2 | | 3 | 4 | |
|  | so nervous that nothing could calm you down? | | | | 0 | | 1 | | 2 | | 3 | 4 | |
|  | hopeless? | | | | 0 | | 1 | | 2 | | 3 | 4 | |
|  | restless or fidgety? | | | | 0 | | 1 | | 2 | | 3 | 4 | |
|  | so restless you could not sit still? | | | | 0 | | 1 | | 2 | | 3 | 4 | |
|  | depressed? | | | | 0 | | 1 | | 2 | | 3 | 4 | |
|  | that everything was an effort? | | | | 0 | | 1 | | 2 | | 3 | 4 | |
|  | so sad that nothing could cheer you up? | | | | 0 | | 1 | | 2 | | 3 | 4 | |
|  | worthless? | | | | 0 | | 1 | | 2 | | 3 | 4 | |
| ***Measure of Processes of Care***  ***“In the past year, to what extent do the people who work with your child…”*** | | | | | | | | | | | | | |
|  |  | not at all | to a very small extent | to a small extent | | to a moderate extent | | to a fairly great extent | | to a great extent | | | to a very great extent |
|  | Help you to feel competent as a parent? | 1 | 2 | 3 | | 4 | | 5 | | 6 | | | 7 |
|  | Provide you with written information about what your child is doing in therapy? | 1 | 2 | 3 | | 4 | | 5 | | 6 | | | 7 |
|  | Provide a caring atmosphere rather than just give you information? | 1 | 2 | 3 | | 4 | | 5 | | 6 | | | 7 |
|  | Let you choose when to receive information and the type of information you want? | 1 | 2 | 3 | | 4 | | 5 | | 6 | | | 7 |
|  | Look at the needs of your ‘whole’ child (e.g. at mental, emotional, and social needs) instead of just at physical needs? | 1 | 2 | 3 | | 4 | | 5 | | 6 | | | 7 |
|  | Make sure that at least one team member is someone who works with you and your family over a long period of time? | 1 | 2 | 3 | | 4 | | 5 | | 6 | | | 7 |
|  | Fully explain treatment choices to you? | 1 | 2 | 3 | | 4 | | 5 | | 6 | | | 7 |
|  | Provide opportunities for you to make decisions about treatment? | 1 | 2 | 3 | | 4 | | 5 | | 6 | | | 7 |
|  | Provide enough time to talk so you don't feel rushed? | 1 | 2 | 3 | | 4 | | 5 | | 6 | | | 7 |
|  | Plan together so they are all working in the same direction? | 1 | 2 | 3 | | 4 | | 5 | | 6 | | | 7 |
|  | Treat you as an equal rather than just as the parent of a patient? | 1 | 2 | 3 | | 4 | | 5 | | 6 | | | 7 |
|  | Give you information about your child that is consistent from person to person? | 1 | 2 | 3 | | 4 | | 5 | | 6 | | | 7 |
|  | Treat you as an individual rather than as a ‘typical’ parent of a child with a disability? | 1 | 2 | 3 | | 4 | | 5 | | 6 | | | 7 |
|  | Provide you with written information about your child’s progress? | 1 | 2 | 3 | | 4 | | 5 | | 6 | | | 7 |
|  | Tell you about the results from assessments? | 1 | 2 | 3 | | 4 | | 5 | | 6 | | | 7 |
|  | Give you information about the types of services offered at the organization or in your community? | 1 | 2 | 3 | | 4 | | 5 | | 6 | | | 7 |
|  | Have information available about your child's disability (e.g. its causes, how it progresses, future outlook)? | 1 | 2 | 3 | | 4 | | 5 | | 6 | | | 7 |
|  | Provide opportunities for the entire family to obtain information? | 1 | 2 | 3 | | 4 | | 5 | | 6 | | | 7 |
|  | Have information available to you in various forms, such as a booklet, kit, video, etc.? | 1 | 2 | 3 | | 4 | | 5 | | 6 | | | 7 |
|  | Provide advice on how to get information or to contact other parents (e.g. organization’s parent resource library)? | 1 | 2 | 3 | | 4 | | 5 | | 6 | | | 7 |
